# Combination of multiple omics techniques for a personalized therapy or treatment selection

**DOI:** 10.3389/fimmu.2023.1258013

**Published:** 2023-09-27

**Authors:** Chiara Massa, Barbara Seliger

**Affiliations:** ^1^ Institute for Translational Immunology, Brandenburg Medical School Theodor Fontane, Brandenburg an der Havel, Germany; ^2^ Institute of Medical Immunology, Martin Luther University Halle-Wittenberg, Halle, Germany; ^3^ Fraunhofer Institute for Cell Therapy and Immunology, Leipzig, Germany

**Keywords:** high throughput technologies, biomarker, cancer, patient stratification, personalized therapy

## Abstract

Despite targeted therapies and immunotherapies have revolutionized the treatment of cancer patients, only a limited number of patients have long-term responses. Moreover, due to differences within cancer patients in the tumor mutational burden, composition of the tumor microenvironment as well as of the peripheral immune system and microbiome, and in the development of immune escape mechanisms, there is no “one fit all” therapy. Thus, the treatment of patients must be personalized based on the specific molecular, immunologic and/or metabolic landscape of their tumor. In order to identify for each patient the best possible therapy, different approaches should be employed and combined. These include (i) the use of predictive biomarkers identified on large cohorts of patients with the same tumor type and (ii) the evaluation of the individual tumor with “omics”-based analyses as well as its *ex vivo* characterization for susceptibility to different therapies.

## 1 Introduction

**Figure 1:**
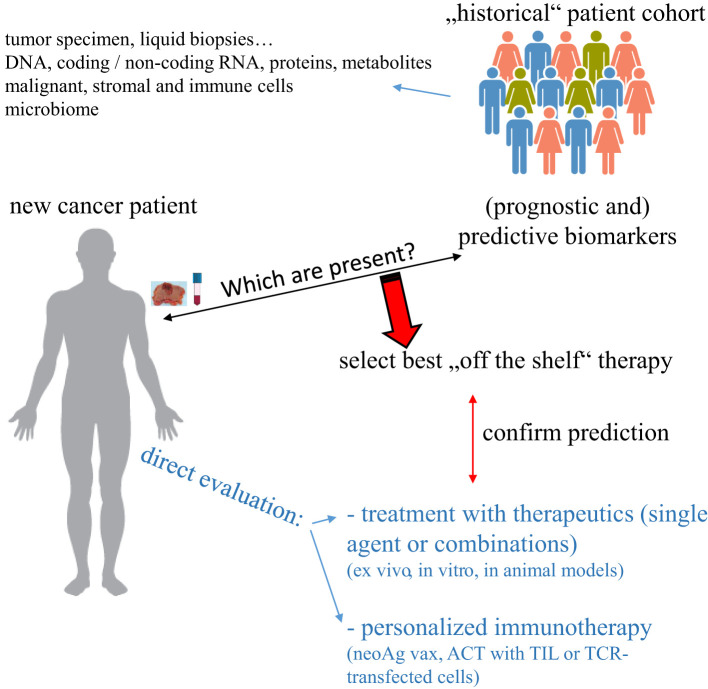
Strategies to select an individually-tailored cancer therapy. Specimen from “new” cancer patients can be evaluated for the expression of biomarkers previously identified to predict response to therapy and/or directly tested *ex vivo* for responsiveness to such therapy. In the case of neoAg as a biomarker, a personalized immunotherapeutic approach can be created.

Cancer is one of the major causes of death worldwide and despite the development of many novel targeted therapies, a high number of patients either do not respond or develop resistance to the treatment. Similar holds true for tumor immunotherapeutic approaches including the treatment with immune checkpoint inhibitors (ICPi), which induce a complete tumor regression, but only in a small number of patients, whose characteristics have not yet been completely understood. Thus, there is an urgent need to determine for each patient the best possible therapy either by identifying biomarkers that can predict response to an available “off the shelf” therapy or by creating an individually-tailored (immune-based) therapy (**Figure 1**). Due to the availability of different high throughput technologies, which are currently also used in clinical research and practice, there exist currently efforts to integrate different omics technologies to advance not only the understanding of the biology of each individual tumor specimen, but also to implement this information e.g. for patients’ stratification and treatment decisions.

In the following paragraphs, we will report on the recent developments in the setting of genomics, transcriptomics, proteomics, microbiomics, metabolomics and immunomics and how they have been beneficial for the management of tumors by providing some examples of clinically relevant discoveries.

## 2 Search for biomarkers for patient stratification

Based on the improvements over the last decade in different “omics” technologies, a huge amount of data was generated from large cohorts of patients with tumors of different histological origin and (sub)types. Large scale genomics, transcriptomics and proteomics analyses have increased our understanding of the (genetic) drivers of cancer and also helped to identify new clinically relevant disease subtypes (1, 2). All these data could be correlated with patients’ clinical characteristics, age, sex, outcome and therapy response in order to identify novel diagnostic, prognostic and predictive markers that will help patients’ stratification to the different therapies currently available.

For example, the improvement in genomic and gene sequencing techniques together with their reduced costs allows deep sequencing not only of different sample types from each patient, including next to tumor lesions also liquid biopsies and stool, but also of multiple types of nucleic acid species, such as DNA and coding as well as non-coding RNA. Moreover, the development of single cell RNA sequencing (scRNAseq) technologies has highlighted a high level of intra-tumoral heterogeneity that was not detected by previous bulk RNA sequence evaluations (3). Sequencing has also been used to identify regulating mechanisms, not only in the form of non-coding RNA species, but also by determining the genome 3D organization, in particular the accessibility of genetic loci to transcription (4).

Furthermore, standard immunohistochemistry (IHC) used for the “pathological/diagnostic” evaluation of tumor samples has been upgraded by different technical approaches, such as the conjugation of antibodies (Ab) with metals or barcodes versus the use of sequential cycles of staining and elution, to multiplex IHC. In such settings, a high number of different Ab can be used on the same tissue slide thereby enabling a deep characterization of the different cell types present within the tumor tissue, which can also be evaluated for their localization and relative spatial distribution and thus their possibility to interact.

The multiplicity of detection has further been extended also to RNA species with different forms of fluorescence *in situ* hybridization (FISH) that allows the identification of more than hundreds of mRNA transcripts/slide at the single cell or almost single cell level (5).

Employing one or a combination of these techniques, a number of “biological read outs” have been evaluated in the search of biomarkers that would allow patients’ stratification with respect to risk and probability to respond to different therapies, thus leading to the selection of an individually tailored therapeutic approach. These biological read outs represent all major hallmarks of cancer (6), ranging from the intrinsic capacity of the transformed cells to proliferate, migrate, survive and rewire their metabolism to the composition of the tumor microenvironment (TME) and the interactions among its different cellular components, namely stromal and immune cells.

### 2.1 Tumor signatures

Neoplastic transformation is mediated by an accumulation of mutations in oncogenes or tumor suppressor genes (7), some of which are shared within the particular tumor type or subtype, whereas others are individual, which might be e.g. the reason, why many patients do not respond to “general tumor type-selected” targeted therapies.

In addition to such inter-patient heterogeneity, therapy resistance is also related to the intra-patient heterogeneity of the tumor. Since a long time it is known that there exists a niche of tumor stem cells with a higher resistance to therapy. Recently, a stemness signature correlating to patients’ risk and low response to ICPi therapy across many tumor types has been described (8). Similarly, a pan-cancer evaluation for prediction of resistance to ICPi identified a malignant cell signature centered on the CDK4/6 pathway, which was associated with the induction of a T cell excluded phenotype (9).

The complexity of the intra-patient heterogeneity of the tumor has been further highlighted by the widespread application of multiplex IHC and scRNAseq, which can identify within the “bulk” tumor mass individual cells expressing characteristics of a tumor subtype distinct from the bulk tumor (10). Such intra-tumor heterogeneity has important consequences in patients with metastatic disease. Indeed, the evaluation of paired samples from primary and lymph node (LN) metastases of breast cancer (BC) patients highlighted discrepancies in the prevalent “molecular subset” between the two locations suggesting that for optimal therapy not only the knowledge of the presence of LN metastases, but also the characterization of their molecular features are required (11).

In addition to bioinformatics analyses of available patients´ data from large cohorts and evaluation of their response to therapy to identify predictive gene signatures, databanks of cell lines and their *in vitro* tested sensitivity to chemotherapeutic drugs are also used to create predicting algorithms of responsiveness [reviewed in (12)]. For example, Geeleher and co-workers employed whole genome expression data obtained from multiple tumor cell lines with known sensitivity to selected drugs to generate a prediction tool allowing the identification of drug sensitivity of tumor from patients in a clinical trial (13). Despite the analyses at the proteomic level are for now far from clinical translation, Tognetti and co-authors were able to identify different signaling pathways in BC cell lines that were able to predict response to specific drugs of patient-derived xenografts (PDX) (14).

### 2.2 Neoantigens

Due to their high proliferation rate, malignant cells accumulate mutations that can lead to the generation of neoantigens (neoAg), i.e. immunogenic peptides encompassing a tumor specific mutation, against which no central tolerance has been created and thus representing optimal targets for immunotherapy (15).

In order to be implemented for therapy, such neoAg have to be identified, which is currently performed by two complementary strategies: (i) at the protein level by elution of peptides associated with the HLA class I and class II molecules on the surface of tumor cell (lines) followed by their identification via mass spectrometry, which leads to the generation of different databanks (16, 17). This approach has been recently expanded to healthy tissue in order to create a reference for a more precise identification of “real” tumor associated neoAg (18). (ii) At the genetic level by comparison of the genomic sequences between malignant and normal cells, which allows the identification of somatic mutations and translocations within the tumor cells. Peptides encompassing such mutations are then subjected to “*in silico*” analysis for the possibility to give rise to epitopes presented via the HLA alleles expressed by the patient (19). In recent years, next to “standard” HLA class I-restricted peptides, these strategies have identified many neoAg-restricted to HLA class II molecules (20, 21) or derived from non-coding sequences (22) leading to the development of new algorithms for their improved identification from sequencing data (23–25). Continuous progresses in artificial intelligence approaches are further improving the capabilities to identify neoAg for clinical application (26).

Some of the identified neoAg are “public” or shared, corresponding to hotspot of mutations present in many tumors within and among different histotypes or derived from viral antigens in viral-driven cancers. In some cases, “off the shelf” therapeutics have been generated for such neoAg, such as transgenic T cell receptors (TCR) or TCR mimics against shared mutations of KRAS (27) and p53 (28) as well as against human papillomavirus (HPV) antigens (29).

However, in most cases, neoAg are private, i.e. specific for each individual tumor, and therefore a personalized vaccine has to be created for each patient. Many different strategies can be implemented, ranging from their direct use as a vaccine in the form of synthetic peptides or of the coding mRNA to their *in vitro* use to load dendritic cells (DC) that will then be employed for vaccination or for *in vitro* expansion of autologous tumor infiltrating lymphocytes (TIL) (30, 31). Such *in vitro* T cell restimulation with the neoAg peptides can be implemented as a screening tool to test the immunogenicity of the predicted epitopes as well as to isolate neoAg-specific T cell clones and their TCR (32). Those TCR sequences could then be cloned and transfected into autologous peripheral T cells to provide a non-exhausted source of neoAg-specific TCR-transgenic effector cells.

Next to the improved identification of possible neoAg, there are also studies to improve their clinical implementation with better strategies for loading DC with polypeptides (33) or optimal spacers for multi-epitope constructs to allow processing into the single peptides (34). Moreover, a genetic and proteomic signature for neoAg-specific CD4^+^ and CD8^+^ T cells has been identified, which could allow the isolation of neoAg-specific T cells from patients’ TIL without the need of previous *in vitro* expansion (35).

### 2.3 Non-coding RNA species

Deep sequencing techniques have identified an array of non-coding RNA species, such as microRNA (miRNA), long non-coding RNA (lncRNA) and circular RNA (circRNA), which are all involved in the regulation of different aspects of malignant transformation (36).

MiRNA are short 20-22 nucleotide RNA sequences that upon binding to complementary regions (seeds) on target mRNA molecules affect their transcription leading in the majority of cases to its inhibition by inducing RNA degradation or inhibiting its translation. Sequencing of patients derived material highlighted multiple miRNA, which could serve as prognostic markers and/or predict treatment response, for example to radiotherapy (RT) and/or tamoxifen in BC patients (37), to RT in prostate cancer (38) and to cisplatin in lung cancer (39).

LncRNA are categorized into different subtypes depending on the chromosomal region from which they are translated and can exert different functions depending on their cellular sublocalization (40). While nuclear located lncRNA are involved in the genomic organization, such as e.g. the inactivation of the second X chromosome in female cells (41), the cytoplasmic lncRNA are involved in post-transcriptional regulation either by acting as a miRNA sponge or by directly interacting with the transcript or with RNA-binding proteins (RBP) (42). Functionally, lncRNA could be involved in all hallmarks of cancer and can therefore be used both as prognostic markers and as therapeutic targets. In multiple myeloma (MM), an array of lncRNA have been associated with resistance to chemotherapy (43). In colorectal cancer (CRC) a lncRNA signature can also predict response to immunotherapy as well as to chemotherapy (44), while in BC linc00665 has been demonstrated to predict response to cisplatin-paclitaxel (45). Evaluation of glioblastoma multiforme identified different immune related lncRNA signatures characterizing different disease subtypes driven by distinct genes and displaying discordant sensitivity to multiple treatments (46).

CircRNA regulates protein translation by different mechanisms, e.g. acting as a sponge for miRNA or protein, but also by interacting with proteins involved in transcription or splicing. In addition, some circRNA can also be translated into proteins (47). Next to being established as diagnostic and prognostic markers in different tumor settings, their use as predictive tool is currently investigated. For example, circ_0026652 could predict response to different targeted treatments in MM (48), whereas in glioblastoma circ-METRN was involved in RT resistance (49). As potential therapeutic target, studies performed with ovarian cancer cell lines indicated a role for circ_0025033 in the resistance to paclitaxel due to the expression of FOXM1 upon inhibition of mir-532-3p (50), whereas circPVT1 protects osteosarcoma from doxorubicin and cisplatin (51, 52) and gastric cancer from paclitaxel (53).

Since both lncRNA and circRNA can compete with miRNA for binding to target mRNA, different studies are currently performed to identify competitive endogenous RNA (ceRNA) networks composed of mRNA, miRNA, lncRNA and circRNA. This will allow to determine the overall effect of all regulatory components at the level of mRNA transcription to improve their diagnostic and/or prognostic value. For example, a prognostic network was identified in acute myeloid leukemia (AML) (54), whereas a ceRNA network was involved in predicting the efficacy of interferon (IFN)-α treatment in hepatocellular carcinoma (55, 56). Similarly, in CRC, a ceRNA signature identified high risk patients, who had also an enhanced sensitivity to different drugs and immunotherapies (57).

Genetic material can also be released by (tumor) cells into the circulation, not only within extracellular vesicles (EV), but also as free molecules. Changes in the repertoire of circulating free DNA(cfDNA) as well as RNA are associated with disease progression and thus studied as diagnostic, prognostic as well as predictive markers (58). For example, serum levels of miR-10b and soluble E-cadherin can predict BC metastases (59), whereas a ceRNA signature in the exosome has been shown to predict response to neoadjuvant chemotherapy in patients with advanced gastric cancer (60).

### 2.4 3D genomic organization

Evaluation of the 3D organization of the genome of transformed cells can provide information on the existence of chromosomal fusion or translocation, which can lead not only to driver mutations and neoAg, but also to changes in the regulation of gene transcription that could affect therapy response. Indeed, mutations in genes such as histone 1 (61) and STAG2 (62) have profound consequences on the 3D genome landscape of the cells by affecting important signaling pathways in tumors. In addition, screening of glioblastoma stem cells from different patients highlighted differences in their 3D genome leading to different signatures and targetable pathways (63). In BC cell lines, changes in the genomic 3D structure during drug treatment or upon acquired resistance were identified, which could possibly help to define new targets for therapy or reversal of resistance (64–66).

Employing “older techniques”, such as FISH and 3D-FISH, differences identified in the translocation between chromosome 9 and 22 in Brc-Abl chronic myeloid leukemia (CML) were associated with the responsiveness to tyrosine kinase inhibitors (TKI), thus correlating the level of nearby chromosome disruption to chemotherapy-responsiveness (67). In a murine BC model, genes in different 3D conformation had a prognostic value for response to endocrine therapy (68)

### 2.5 Metabolism

Alterations in the tumor metabolism are one hallmark of cancer that not only intrinsically allow malignant cells to proliferate and survive, but also help to establish an immunosuppressive TME, thus further favoring tumor development through immune escape (69). The tumor associated metabolism has been characterized at the level of the mutational profile and the expression of metabolic genes within tumor specimens, by the characterization of metabolites in liquid biopsies via mass spectrometry or directly within the patients by using specific reagents for PET-CT (70) as well as other emerging techniques (71).

General differences in the expression of metabolic genes allowed to stratify patients with ovarian cancer into high and low risk patients and could also predict an enhanced response to different chemotherapies (72). In addition, a 7 metabolism-gene signature identified in BC and further validated in melanoma and urothelial epithelial cancer was stratifying patients for outcome and therapy response (73). Enhanced biosynthesis of nicotinamide adenine dinucleotide (NAD^+^), an abundant metabolite that plays a key role in cellular homeostasis, stemness and immune response (74), not only discriminates healthy versus BC tissue, but can also identify a subgroup of patients with a worse prognosis (75). Moreover, since patients with a high NAD^+^ biosynthesis have a higher immunogenicity as well as an increased immune suppression, this score might be implemented to select patients with enhanced responsiveness to immunotherapies, such as ICPi (75).

Other studies focused on specific metabolic pathways in order to stratify patients. The most known alteration in the tumor metabolism is the Warburg effect, namely the prevalent usage of anaerobic glycolysis to degrade glucose even in the presence of oxygen (76). In such context, Sun and co-authors identified a lactate related signature in renal cell carcinoma (RCC) patients, which can predict overall survival (OS) (77).

In light of the important role of lipids for signaling as well as for membrane formation, Zhu and coauthor analyzed specimens from bladder cancer identifying a gene signature with 11 lipid-related genes that was able not only to stratify patients better than the currently used system based on clinical characteristics, but also to predict response to immunotherapy (78). In cervical cancer, a signature based more specifically on fatty acid metabolism identified high risk patients (79), whereas the sphingolipid metabolism in association with hypoxia stratified patients with pancreatic ductal adenocarcinoma (80).

Amino acids and their metabolism have also been evaluated. In different tumor histotypes, the presence of specific free amino acids in biological fluids can be used as an early diagnostic marker for tumor development as well as for more subtle patients’ stratification regarding grading and outcome (81, 82). In ovarian cancer, a score based on the expression of genes associated with the adenosine metabolism was able to identify patients with a shorter survival and with a (predicted) lower sensitivity to different chemotherapeutics (83), while in lung adenocarcinoma, a signature associated with a low glutamine metabolism identified low-risk patients, which respond to immunotherapy (84)

### 2.6 Microbiome

The studies performed during the last decade demonstrated that the different host-intrinsic microorganisms composing the microbiome are an important component of the human body, which influence many different functions, ranging from the cellular metabolism to the immune response (85). Thus, alterations in the microbiome composition can have consequences on disease development and therapy response. With the widespread implementation of shotgun metagenomics or 16S rRNA sequencing to evaluate the different species composing the microbiota, a huge amount of information on the microbiome of patients with different tumor types and responding or not to different therapies has been generated and investigated for biomarkers, which could be used for patient stratification as well as for possible therapeutic approaches to improve treatment outcomes in cancer (86).

Whereas the first studies analyzed the gut microbiome, which is the most abundant in the host and easy to sample, the focus has now also moved to the investigation of the intra-tumoral microbiota. Indeed, for all the major tumor types, also from soft tissue with no direct contact with the outer world (87), the presence of intra-tumoral bacteria, viruses and archea has been demonstrated, which is different to the corresponding normal tissue microbiome, indicating a “non-random” mechanism of accumulation. Indeed, evaluation of the spatial distribution of the microorganisms within the tumor lesions indicated a specific accumulation into niches enriched of immunosuppressive cells and depleted of T cells, thus underlying an active interaction with the components of the TME (88). Also for the intra-tumoral microbiome, there is a high level of inter-patient heterogeneity (89).

The determination of the composition of the gut microbiota was used for general prognosis as well as for the prediction of patients’ responsiveness to systemic therapies, such as chemotherapies and immunotherapy, thereby linking the response to treatment with the presence or absence of different species (90–92). In contrast, the response to local therapy, such as RT, has been associated with the intra-tumoral, but not with the gut microbiome (87)

In some cases, the mechanisms responsible for the correlation with the patients’ outcome were also identified. For example, different gamma-proteobacteria strains can protect CRC from gemcitabine chemotherapeutics by directly metabolizing it into its inactive form (93), whereas *Fusobacterium nucleatum* promotes chemo-resistance in this disease by activating the autophagy pathway of tumor cells and thus protecting them from apoptosis induction (94). The opposite mechanism, a reduction of tumor autophagy due to accumulation of reactive oxygen species, is responsible for the protective role of the microbiota metabolite indole-3-acetic acid in pancreatic cancer (95). Instead, the promotion of tumor cell death by pyroptosis is responsible for the enhanced response to immunotherapy of triple negative breast cancer (TNBC) patients with higher intra-tumoral levels of the microbial metabolite trimethylamine N-oxide (96). Another mechanism, by which the microbiota can influence tumor development, is the neoAg presentation via HLA surface antigens. Indeed, characterization of peptides eluted from melanoma metastasis identified many HLA class I as well as class II epitopes derived from intracellular pathogens, which could also be recognized by the patients´ TIL (97).

In addition to its prognostic and predictive role, the microbiota could also be used therapeutically to improve therapy response via fecal transfer from healthy donors or from patients responding to the same therapy (98).

### 2.7 Immunomonitoring

Due to the central role of the immune system not only in the natural immune-surveillance against malignant transformation, but also as a target and mode of therapy, a large array of comparison of the immune system in cancer patients versus healthy individuals as well as in responder and non-responder patients was performed using different technologies and different biomaterials. Immune cells have been identified at the protein level by direct staining with Ab using flow cytometry and (multiplex) IHC or identified within bulk transcriptomic data using different algorithms, such as CIBERSORT (99) or directly by scRNAseq.

Screening can be done on both tumor and liquid biopsies. The first has the advantage of representing the site of the disease and thus the presence and location of the immune cells is highly informative. Since it requires surgery, it is mainly used for diagnostic purposes and not for longitudinal evaluation, whereas liquid biopsies, such as blood samples or lavages, are easier to obtain at multiple time points, but only represent the systemic and not the local composition, spatial distribution and status of the immune system.

Initial markers for patients’ stratification using tumor tissues were the evaluation of TIL numbers followed by the development of the immunoscore, where the cell subtypes and their broad location (margin versus tumor center) acquired importance (100). With the improvement of multiplex IHC and of software for data analysis, the spatial distance between different cell types (101, 102), their organization in particular cellular neighborhoods (103), TME archetypes (104) or tertiary lymphoid structure (TLS) (105) could be determined and correlated with response to therapy. Since PD-L1 expression within the tumor is not a good predictor for response to ICPi, many evaluations have focused on its receptor. Due to the opposing effects of PD1 signaling in CD8^+^ effector T cells and in regulatory T cells (Treg), the relative frequency of CD8^+^ T cells and Treg expressing PD1 within the TME was found to affect response to ICPi (106). Moreover, high levels of PD1^+^ Treg were correlated with the hyper-progressor phenotype of patients treated with anti-PD1 Ab (107).

Analysis of the immune cell repertoire in peripheral blood by multicolor flow cytometry or mass cytometry (CyTOF) allow to detect the phenotype of effector cells or the presence of immunosuppressive populations as well as their function/activity. Regarding immunosuppressive subsets and their soluble mediators, a score based on the presence of different myeloid cells identifies melanoma patients with a worse prognosis (108), whereas the amount of IL-13 in the sera of patients with diffuse large B cell lymphoma lesions represents a signature of Treg and is associated with a poor OS (109). An enhanced neutrophil to lymphocyte ratio, which since long time is correlated with a worse patients’ prognosis (110), is accompanied by a reduced response to anti-PD1 in non-small lung cell cancer (111). Similarly, higher basophil counts in gastric cancer are associated with a low response to anti-PD1 Ab in combination to chemotherapy, but not to chemotherapy alone (112). In contrast, enhanced starting levels of “immunostimulatory” monocytes (113) and a functional CD4^+^ T cell compartment (114) predict therapy response. In addition to these “baseline” markers, which are needed for initial patient stratification to therapy, there is also the need of markers during treatment that confirm response or indicate the requirement of a therapy change or optimization due to unresponsiveness or resistance development. In addition to blood evaluation at patient´s first presentation for therapy stratification, immunomonitoring can also be performed longitudinally, during therapy, in order to determine responsiveness to the therapy. For example, the presence of proliferating T cells (i.e. expressing Ki-67) is predictive for a good clinical outcome and therapy response in lung cancer patients (115), whereas in melanoma T cell proliferation has to be normalized to the tumor burden in order to significantly discriminate responding patients (116). Similar discrepancies among different tumor types were found regarding the clonality of the immune response, with a more clonal or a more diverse TCR repertoire correlating with response to ICPi in different tumor types (117).

Based on the availability of databases with clinical as well as RNAseq data from cancer patients undergoing immunotherapy with ICPi, many different immune-related genetic signatures have been identified that correlated with the response, such as the T cell-inflamed signature (118), the adaptive immune response associated with a pro-tumorigenic inflammation ratio (119) and an ICPi responsive B cell cluster signature (120). Moreover, additional signatures focusing on all aspects of the TME have been generated for better patients’ stratification (121). A different strategy used tumor cells obtained from patients’ material co-cultured *in vitro* with limiting dilution of the autologous, *in vitro* expanded TIL in order to identify a “tumor undergoing T cell attack” signature, which included many components involved in IFNγ signaling and allowed the prediction of the clinical outcome to ICPi in multiple tumor types (122).

Biomarkers predicting response to therapy are required also for other immunotherapeutic interventions, such as for example adoptive cell therapy (ACT). Indeed, due to its personalized nature, implementing autologous TIL expanded *in vitro* or the autologous T cells transfected with chimeric antigen receptor (CAR), the therapeutic agent of ACT is a variable on its own, which has to be optimized for optimal usage. To this aim, the final expanded cell products have been characterized in depth and correlated to the patients’ outcome in order to identify T cell phenotypes and subpopulations (123–125) or expression pattern (126, 127), which are positively or negatively associated with the clinical response and could thus be implemented to improve the efficacy of future preparations. In line with the longitudinal evaluation of response to ICPi, also blood samples from patients undergoing ACT have been analyzed to identify (early) predictive markers of response that might allow possible therapy changes or improvement by e.g. implementing combinations with other treatments. Upon CAR T cell transfer, expansion of the injected cells did not correlate with response (128, 129), while enhanced levels of different subsets such as CD4^+^ and CD8^+^ CAR cells expressing CD57 and T-bet (129), or CD4^+^ CAR T cells expressing PD1 and LAG3 as well as lower levels of CD8^+^ CAR T expressing CD107 (128) did predict response.

## 3 Patient derived material for therapy selection

Whereas most of the approaches described above aim to identify biomarker(s)/signature(s) able to predict the responsiveness of a tumor specimen to different treatments in order to select the optimal therapy or combination thereof, tumor cells from the patient specimen can also be directly tested *ex vivo* to evaluate or confirm the predicted susceptibility to available treatments. The experimental settings, which have been implemented to study the tumor development and therapy response with established tumor cell lines, have been adapted for the use of patient derived material. In the following paragraphs, we present those different approaches together with their advantages and limitations for the implementation in personalized medicine and provide some examples of their clinical application.

### 3.1 Culture of tissue slices/pieces

For the development of slice cultures, tumor material derived from surgical resection is directly cut into pieces or slices, which are then cultured in the presence or absence of the different treatments to be evaluated. These include not only chemotherapeutics or targeted drugs, but also immune-based therapies, such as ICPi, since the full cellular repertoire with its local distribution is preserved within the slice for at least a few days up to 2 weeks without undergoing (excessive) spontaneous cell death (130, 131).

This procedure has been successfully applied to tumors from the liver (132), pancreas (133, 134), stomach and gastroesophageal junction (135), lung (130), prostate and bladder (131) and from hepatic metastasis of CRC (136). At different time points, the slices can be evaluated to determine the level of tumor cell death and when immunotherapeutics were applied to investigate the proliferation and/or relocalization of immune cells (130, 132). Despite the short turn-around time required for the read out and the retention of the tumor composition and structure, a strong limitation of this technique is associated to its low throughput, since the number of conditions that can be evaluated is restricted by the size of the resection specimen and thus the amount of slices, which could be generated.

### 3.2 *In vitro* culture of tissue digested material

In order to obtain a “never ending source” of malignant cells, tumor specimens have been mechanically and enzymatically digested in order to obtain tumor cell lines that could be then tested *in vitro*. Whereas in the last century, pure tumor cell lines have been obtained and used for drug susceptibility screening in 2D monolayer, nowadays the attempt is to grow tumor cells in 3D spheroids, which better resemble the *in vivo* situation with the formation of concentration gradients and the presence of the physical restrain of a solid tumor mass (137). To mimic more the *in vivo* situation, 3D organoids are currently generated, which include tumor cells as well as stromal cells, like cancer associated fibroblasts (CAF), which might be involved in therapy resistance of the tumor *in vivo*. Different protocols have been established for organoid cultures of different tumor histotypes (138), which were also improved to allow high throughput analysis (139–141). Despite such organoids do not retain the immune cell infiltrate, autologous peripheral blood mononuclear cells (PBMC) as well as TIL can be co-cultured with the organoids in order to evaluate responsiveness to different (immuno)therapeutic approaches (142).

Different cases of highly successful selection of therapy upon screening with organoid have been recently reported in patients with ovarian and lung cancer (143, 144).

### 3.3 Xenograft setting

In order to physiologically reproduce the *in vivo* conditions of tumor growth, human tumors have been transplanted into immune-deficient mice as PDX, which could be evaluated in an *in vivo* setting for susceptibility to chemotherapy. In order to be able to evaluate also immunotherapeutic approaches, new strains of immune-deficient mice have been developed in order to allow a better engraftment of human immune cells, such as hematopoietic CD34^+^ stem cells (145, 146) and autologous PBMC (147), or to promote the survival of TIL present within the tumor specimen (148).

Despite being highly relevant, these murine models are more prone to be used for mechanistic and functional studies and retrospective analysis to understand why cancer patients are therapy responders or developed resistances than for direct selection of personalized therapy. Indeed, the time length required for their establishment is an obstacle to their implementation for high risk patients and highly aggressive tumors. Despite that, a combination of organoid and PDX was successfully used for personalized therapy selection for a patient with gallbladder cancer (149). Similarly, for a patient with an urothelial bladder cancer with HRAS mutation, a combination of scRNAseq and PDX identified an upregulation of PD-L1 on chemoresistant cells, thus leading to the treatment of this patient with the anti PD-L1 Ab atezolizumab (150).

Zebrafish has mainly been used as a model to study tumor development but is currently also implemented in the context of personalized therapy. It has many advantages over the conventional murine models including a shorter time required for generation of results and of genetically modified species, lower costs and due to the transparency of its cells an easier evaluation of growing tumor cells than in murine models (151). Establishment of a high throughput system to image and quantify tumor growth further amplify the potential usefulness of this model system (152).

Not only solid tumors, such as melanoma (153), BC (154) and gastric cancer (155), but also different hematopoietic malignancies, such as B cell precursor acute lymphoblastic leukemia (156), CML and AML (157), have been successfully transplanted into zebrafish giving rise to zebrafish PDX (zPDX). The system does not only allow evaluation of responsiveness to chemotherapy, but has also been evaluated to test sensitivity to radiotherapy (158) and its possible enhancement by combination with other drugs, such as metformin (159). Moreover, zPDX are also being implemented to evaluate susceptibility to immunotherapy either in the form of CAR T cells (160, 161) or of retargeting bispecific antibodies, which are injected together with autologous PBMC (162). In this context, it is noteworthy that currently two clinical trials in patients with pancreatic ductus adenocarcinoma (PDAC) (163) and CRC (164) are performed using the zPDX setting for the selection of the patients’ optimal therapy.

## 4 Outlook

As described above many progresses have been made in the identification of blood- and tissue-based biomarkers either for patients’ stratification to therapy or to determine their responsiveness to it. In addition, new possible therapeutic targets have also been characterized. Despite most of the approaches reported used only one “omic” technique, there is increasing evidence that for the selection of the best possible therapeutic option for each individual patient, multiple features of the tumor have to be evaluated since its development is influenced by genetic, epigenetic and environmental factors. Combination of data from multiple “omics” profile from a single patient will provide powerful tool to generate a holistic view of molecular, metabolic and immunological effects, which can be used to predict response to therapy. Different strategies based on machine learning have been developed during the last years to perform data integration and have recently been reviewed in various articles (165–168). However, it is noteworthy and has to be taken into account that “omics” data are fundamentally different. While genetic variation data are discrete and static, RNAseq measurements, metabolic profiling or immuno-monitoring are continuous, time dependent, but on the other hand could provide longitudinal information.

Despite all these difficulties, preliminary studies have demonstrated the feasibility to integrate data obtained from different techniques in order to identify the best possible therapy for the patients within a clinical timeframe (169).

## Author contributions

CM: Writing – original draft, Writing – review & editing. BS: Writing – review & editing.

## Glossary

**Table:**

Adoptive cell therapy	ACT
acute myeloid leukemia	AML
antibody	Ab
breast cancer	BC
cancer associated fibroblast	CAF
chimeric antigen receptor	CAR
circulating free DNA	cfDNA
chronic myeloid leukemia	CML
circular RNA	circRNA
colorectal cancer	CRC
dendritic cell	DC
extracellular vesicle	EV
fluorescence *in situ* hybridization	FISH
immune checkpoint inhibitor	ICPi
interferon	IFN
immunohistochemistry	IHC
lymph node	LN
long non-coding RNA	lncRNA
microRNA	miRNA
multiple myeloma	MM
neoantigen	neoAg
pancreatic ductus adenocarcinoma	PDAC
patient-derived xenograft	PDX
peripheral blood mononuclear cell	PBMC
radiotherapy	RT
renal cell carcinoma	RCC
RNA-binding protein	RBP
single cell RNA sequencing	scRNAseq
T cell receptor	TCR
tertiary lymphoid structure	TLS
triple negative breast cancer	TNBC
tumor infiltrating lymphocyte	TIL
tumor microenvironment	TME
tumor mutational burden	TMB
regulatory T cell	Treg
tyrosine kinase inhibitor	TKI
zebrafish PDX	zPDX
